# Supplementary material for: A family of lead clusters with precious metal cores
Source: Nat Commun. 2020 Jul 10;11:3477. doi: 10.1038/s41467-020-17187-4 (PMC7351731; doi:10.1038/s41467-020-17187-4)
Supplement: Supplementary file 1 — Supplementary Information [file 41467_2020_17187_MOESM1_ESM.pdf]

Supplementary Information

A family of lead clusters with precious metal cores

Shu *et al*.

Contents

Supplementary Methods

Section 1: Methods and Materials 4

X-ray Diffraction. 4

Electrospray Ionization Mass Spectrometry (ESI-MS) 5

Energy Dispersive X-ray (EDX) Spectroscopic 5

Section 2: Crystallographic Supplementary inofrmation 6

Supplementary Table 1. X-ray measurements and structure solution of compound **1**, **2** and **3**. 6

Supplementary Figure 1. Crystals of 1, 2 and 3. 7

Supplementary Table 2. Selected interatomic distances (in Å) of **1** in both the X-ray and DFT-optimized geometries. 7

Supplementary Table 3. Selected interatomic distances (in Å) in the X-ray structure of compound **2**. 8

Supplementary Table 4. Selected interatomic distances (in Å) in the X-ray geometry of compound **3**. 10

Supplementary Figure 2. Asymmetric unit of compound **1** with the cluster fragment. 13

Supplementary Figure 3. Unit cell of compound **1** 13

Supplementary Figure 4. Asymmetric unit of compound **2** with the cluster fragment. 13

Supplementary Figure 5. Unit cell of compound **2** 14

Supplementary Figure 6. Asymmetric unit of compound **3** with the cluster fragment. 14

Supplementary Figure 7. Unit cell of compound **3**. 15

Supplementary Figure 8. The decomposition and recombination of $[Au_8Pb_{33}]^{6-}$ and $[Au_{12}Pb_{44}]^{8-}$ clusters. 16

Supplementary Table 5. Summary of structural data for [M@Pb_{11}] units (M = Ag, Au). 16

Section 3: Electrospray Ionization Mass Spectrometry (ESI-MS) Analysis 17

Supplementary Figure 9. ESI-MS of the reaction of K_4Pb_9 with Au(Mes)PPh_3 in negative-ion mode. 17

Supplementary Figure 10. Experimental (black) and simulated (red) spectra of the fragments $[AuPb_{10}]^-$ and $[AuPb_{11}]^-$. 17

Supplementary Figure 11. Experimental (black) and simulated (red) spectra of the fragments $[AuPb_{12}]^-$ and [K([2.2.2]crypt)]$[Au_2Pb_{11}]^-$. 19

Supplementary Figure 12. ESI-MS of the reaction of K_4Pb_9 with $(AgMes)_4$ in negative-ion mode. 19

Supplementary Figure 13. Experimental (black) and simulated (red) spectra of the fragments $[AgPb_{10}]^-$ and $[AgPb_{11}]^-$. 20

Supplementary Figure 14. Experimental (black) and simulated (red) spectrums of the fragment, $[AgPb_{12}]^-$. 22

Section 4: Energy Dispersive X-ray (EDX) Spectroscopic Analysis 23

Supplementary Figure 15. EDX analysis of complex **1** (K, Ag, Pb), **2** and **3** (K, Au, Pb). 23

Section 5: Quantum chemical studies 24

Supplementary Table 6. Optimized structures and total energies of the M^+ ions and the fragments $[M@Pb_{11}]^{3–}$, and $[M@MPb_{11}]^{2–}$ (M = Ag, Au). 21

Supplementary Table 7: Energy decomposition analysis (EDA) for the interaction between M^+ and $[M@Pb_{11}]^{3–}$, M = Ag and Au........ 23

Supplementary Discussion

Supplementary Figure 16. Secondary Au…Pb distances in the crystal structures (blue) and the D_{2d}-symmetrized structure (red). 27

Supplementary Figure 17. Total energies of structures with Au and Pb permuted into different positions. 24

Supplementary Table 8. Total interaction energies and their components (in eV) for the fragmentation of $[Au_{12}Pb_{44}]^{8–}$ into $([Au@Pb_{11}]^{3–})_4$ + $[Au_8]^{4+}$ 24

Supplementary Table 9. Nalewajski-Mrozek Bond orders 28

Supplementary References 28

Supplementary Methods

Section 1: Methods and Materials

All manipulations and reactions were performed under a nitrogen atmosphere using standard Schlenk-line or glove box techniques. K_4Pb_9 was synthesized by heating a mixture of the stoichiometric elements at 400 °C for 72h in a sealed niobium tube. $Au(Mes)PPh_3$ and $(AgMes)_4$ (Mes = 2,4,6-trimethyl) was synthesized according to the reported literature.[1,2] 4,7,13,16,21,24-Hexaoxa-1,10-diazabicyclo[8.8.8]hexacosane ([2.2.2]crypt, Sigma-Aldrich 98%) were dried under vacuum for several hours and transfer to glove box for use. Pyridine (Aldrich, 99.8%) and toluene (Aldrich, 99.8%) were distilled by sodium in a nitrogen atmosphere and stored in a glove box prior to use. Ethylenediamine (en) (Aldrich, 99%) and Dimethylformamide (DMF) (Aldrich, 99.8%) was freshly distilled by CaH_2 prior to use.

X-ray Diffraction.

Suitable single crystals of **1**, **2** and **3** were selected for X-ray diffraction analyses. Crystallographic data were collected on Rigaku XtalAB Pro MM007 DW diffractometer with graphite monochromated Cu Kα radiation (λ = 1.54184 Å). Structures were solved using direct methods and then refined using SHELXL-2014 and Olex2 [3-5] to convergence, in which all the non-hydrogen atoms were refined anisotropically during the final cycles. All hydrogen atoms of the organic molecule were placed by geometrical considerations and were added to the structure factor calculation. Given the difficulties in distinguishing Au and Pb in the crystallographic experiment, we note here that the coordination environments of the sites assigned to Au and Pb in 2 and 3 are very different. The sites assigned to Pb pentagonal pyramidal, a very typical geometry for a tetrel element bearing a lone pair, and indeed the $M@Pb_{11}$ and $M@Pb_{12}$ motifs are well established in the literature. The sites assigned as Au, in contrast, are coordinated from both sides, a more typical environment for a spherically symmetric d^{10} cation. To strengthen these qualitative arguments, we have compared the computed energies of the proposed structure of the cluster anion in 3 ($[Au_{12}Pb_{44}]^{8-}$) with alternatives where the positions of Au and

Pb atoms are permuted. These permutations are done four atoms at a time to conserve the D_{2d} symmetry. Permuting an Au atom in the endohedral site with Pb destabilizes the cluster by ~4 eV per atom, while permuting an Au atom in the surface site costs ~2.5 eV per atom (the numbers depend slightly on the position from where the Pb atom was taken). Permuting an Au atom in one of the capping sites (Au8-11), in contrast, destabilizes the cluster by only ~0.25 eV per substitution. This reflects the closer similarity in the enviroments – if the secondary interactions are ignore then the Pb sites and the capping Au sites are both pyramidal. Nevertheless, the calculations indicate that the most stable permutation of atoms in the crystal structure is as presented in Figures 3 and 4 of the main text.

Electrospray Ionization Mass Spectrometry (ESI-MS)

ESI-MS of the DMF reaction mixture were performed on Agilent Technologies ESI-TOF-MS (6230) in negative-ion mode. The spray voltage was 5.48 kV and the capillary voltage was 30 V. The capillary temperature was 300°C. All samples were prepared under a nitrogen atmosphere.

Energy Dispersive X-ray (EDX) Spectroscopic

EDX analysis were performed using a scanning electron microscope (FE-SEM, JEOL JSM-7800F, Japan). Data acquisition was performed with an acceleration voltage of 15 kV and an accumulation time of 60 s.

Section 2: Crystallographic Supplementary Information

Supplementary Table 1. X-ray measurements and structure solution of compound **1**, **2** and **3**.

Compound	**1**	**2**	**3**
Empirical formula	$C_{55}H_{112}N_7O_{18}K_3AgPb_{11}$	$C_{110}H_{224}Au_8K_6N_{14}O_{36}Pb_{33}$	$C_{149}H_{293}Au_{12}K_8N_{17}O_{48}Pb_{44}$
Formula weight	3663.77	10968.65	14884.18
Temperature /K	100	100	100
Wavelength /Å	1.54184	1.54184	1.54184
Crystal system	triclinic	monoclinic	triclinic
Space group	*P*-1	$P2_1$/n	*P*-1
a /Å	15.1373(2)	17.9414(2)	23.3059(3)
b /Å	16.1785(2)	59.9524(5)	24.1892(3)
c /Å	21.2671(3)	21.1936(2)	26.7122(3)
α/°	96.1805(11)	90	92.6235(9)
β/°	91.7462(11)	112.1180(10)	91.1460(9)
γ/°	111.1044(13)	90	91.1723(10)
V /$Å^3$	4817.20(12)	21118.9(4)	15036.8(3)
Z	2	4	2
ρ_{calc} /g·cm^{-3}	2.527	3.450	4.418
$\mu(Cu_{K\alpha})$ /mm^{-1}	39.687	61.526	68.114
F(000)	3284.0	18896	17615
2Θ range /°	3.14 to 73.71	2.37 to 73.86	2.49 to 74.17
Reflections collected / unique	56367/18834	109926/41216	161687/58602
Data / restraints / parameters	18834/6/857	41216/110/1827	58602/20/1450
R_1/wR_2 (I>2σ(I))[a]	0.0538/ 0.1463	0.0684/0.1728	0.1506/0.4184
R_1/wR_2 (all data)	0.0658/ 0.1524	0.0837/0.1806	0.1706/0.4329
GooF (all data)[b]	1.067	1.076	1.830
Data completeness	0.967	0.963	0.957
Max. peak/hole /e^-·$Å^{-3}$	6.88/-2.91	6.517/-2.775	11.156 /-5.271

[a] R_1= = $\sum||F_o|-|F_c||/\sum|F_o|$; $wR_2 = \{\sum w[(F_o)^2-(F_c)^2]^2/\sum w[(F_o)^2]^2\}^{1/2}$

[b] GooF = $\{\sum w[(Fo)^2-(Fc)^2]^2/(n-p)\}^{1/2}$

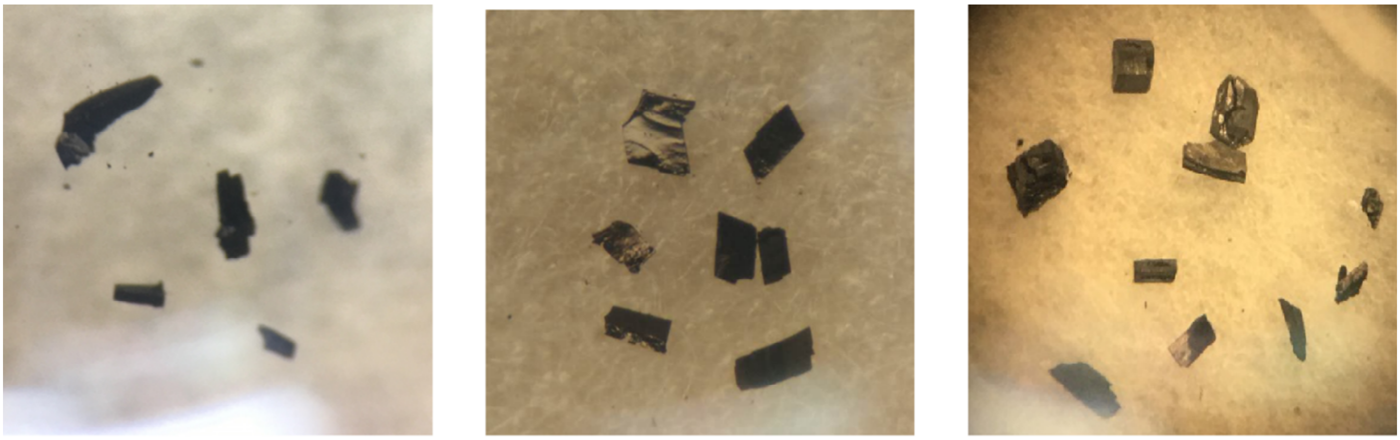

(a) (b) (c)

Supplementary Figure 1: **Crystals of 1, 2 and 3** (a) black block crystals of $[K([[2.2.2]crypt)]_3[Ag@Pb_{11}]\cdot 0.5en$, 1. (b) black strip crystals of $[K([2.2.2]crypt)]_6[Au_8Pb_{33}]\cdot en$, 2. (c) black block-like crystals of $[K([2.2.2]crypt)]_8[Au_{12}Pb_{44}]$, 3.

Supplementary Table 2. Selected interatomic distances (in Å) of 1 in both the X-ray and DFT-optimized geometries.

	X-ray	Calculated		X-ray	Calculated
Pb(1)-Pb(2)	3.2627(8)	3.36	Pb(6)-Pb(11)	3.1808(7)	3.24
Pb(1)-Pb(3)	3.2322(8)	3.36	Pb(6)-Pb(10)	3.1525(6)	3.24
Pb(1)-Pb(4)	3.2575(8)	3.36	Pb(7)-Pb(8)	3.2156(7)	3.28
Pb(1)-Pb(5)	3.2479(9)	3.36	Pb(8)-Pb(9)	3.1649(7)	3.28
Pb(1)-Pb(6)	3.2246(7)	3.36	Pb(9)-Pb(10)	3.1784(7)	3.28
Pb(2)-Pb(3)	3.2491(7)	3.38	Pb(10)-Pb(11)	3.1816(7)	3.28
Pb(2)-Pb(6)	3.3076(7)	3.38	Pb(7)-Pb(11)	3.1961(8)	3.28
Pb(2)-Pb(7)	3.1476(8)	3.24	Ag(1)-Pb(1)	3.0173(9)	3.08
Pb(2)-Pb(11)	3.1661(7)	3.24	Ag(1)-Pb(2)	3.0976(9)	3.18
Pb(3)-Pb(4)	3.3009(8)	3.38	Ag(1)-Pb(3)	3.0845(9)	3.18
Pb(3)-Pb(7)	3.1657(8)	3.24	Ag(1)-Pb(4)	3.1176(9)	3.18
Pb(3)-Pb(8)	3.1655(7)	3.24	Ag(1)-Pb(5)	3.1010(9)	3.18
Pb(4)-Pb(5)	3.2746(8)	3.38	Ag(1)-Pb(6)	3.0848(9)	3.18
Pb(4)-Pb(8)	3.1488(8)	3.24	Ag(1)-Pb(7)	3.0079(9)	3.11
Pb(4)-Pb(9)	3.1820(8)	3.24	Ag(1)-Pb(8)	3.0150(9)	3.11
Pb(5)-Pb(6)	3.2424(7)	3.38	Ag(1)-Pb(9)	3.0073(9)	3.11
Pb(5)-Pb(9)	3.1776(7)		Ag(1)-Pb(10)	3.0119(9)	3.11
Pb(5)-Pb(10)	3.1722(8)		Ag(1)-Pb(11)	3.0293(9)	3.11

Supplementary Table 3. Selected interatomic distances (in Å) in the X-ray structure of compound 2.

	X-ray		X-ray
Pb(1)-Au(1)	2.9632(12)	Pb(18)-Pb(22)	3.1854(13)
Pb(1)-Pb(4)	3.2354(13)	Pb(18)-Pb(19)	3.2273(13)
Pb(1)-Pb(3)	3.2372(15)	Pb(19)-Au(5)	3.0042(12)
Pb(1)-Pb(5)	3.2425(15)	Pb(19)-Au(2)	3.1521(13)
Pb(1)-Pb(6)	3.2448(13)	Pb(19)-Pb(20)	3.2874(13)
Pb(1)-Pb(2)	3.2556(15)	Pb(19)-Au(8)	3.3589(12)
Pb(2)-Au(1)	3.0196(12)	Pb(20)-Au(5)	3.0324(11)
Pb(2)-Pb(3)	3.1901(14)	Pb(20)-Pb(21)	3.3054(12)
Pb(2)-Pb(6)	3.2024(17)	Pb(20)-Au(2)	3.3416(12)
Pb(2)-Pb(8)	3.2899(14)	Pb(20)-Au(6)	3.4098(12)
Pb(2)-Pb(7)	3.3125(14)	Pb(20)-Au(7)	3.4936(12)
Pb(3)-Au(1)	2.9813(12)	Pb(20)-Pb(30)	3.5945(11)
Pb(3)-Pb(8)	3.2453(14)	Pb(21)-Au(5)	3.0434(12)
Pb(3)-Pb(9)	3.2577(13)	Pb(21)-Au(2)	3.1324(12)
Pb(3)-Pb(4)	3.2665(14)	Pb(21)-Pb(22)	3.1850(12)
Pb(4)-Au(1)	3.0131(12)	Pb(21)-Au(7)	3.2988(12)
Pb(4)-Pb(5)	3.2295(14)	Pb(22)-Au(5)	2.9561(12)
Pb(4)-Pb(9)	3.2395(14)	Pb(22)-Au(2)	3.1241(11)
Pb(4)-Pb(10)	3.2767(12)	Pb(23)-Au(3)	2.9639(11)
Pb(5)-Au(1)	2.9925(13)	Pb(23)-Pb(25)	3.2274(15)
Pb(5)-Pb(6)	3.2198(15)	Pb(23)-Pb(26)	3.2438(13)
Pb(5)-Pb(11)	3.2362(13)	Pb(23)-Pb(24)	3.2511(16)
Pb(5)-Pb(10)	3.3097(14)	Pb(23)-Pb(27)	3.2543(16)
Pb(6)-Au(1)	3.0112(12)	Pb(23)-Pb(28)	3.2612(13)
Pb(6)-Pb(11)	3.2729(13)	Pb(24)-Au(3)	3.0123(12)
Pb(6)-Pb(7)	3.3057(14)	Pb(24)-Pb(28)	3.2219(16)
Pb(7)-Au(4)	2.9937(11)	Pb(24)-Pb(25)	3.2547(12)
Pb(7)-Pb(11)	3.1803(14)	Pb(24)-Pb(29)	3.2558(13)
Pb(7)-Au(1)	3.1886(11)	Pb(24)-Pb(33)	3.2811(12)
Pb(7)-Pb(8)	3.1932(14)	Pb(25)-Au(3)	3.0245(12)
Pb(7)-Pb(18)	3.5706(13)	Pb(25)-Pb(26)	3.2203(14)
Pb(7)-Pb(22)	3.6845(13)	Pb(25)-Pb(30)	3.2601(12)
Pb(8)-Au(4)	2.9425(13)	Pb(25)-Pb(29)	3.2694(12)
Pb(8)-Au(1)	3.1285(13)	Pb(26)-Au(3)	3.0068(12)
Pb(8)-Pb(9)	3.2538(14)	Pb(26)-Pb(27)	3.2324(14)
Pb(9)-Au(4)	3.0505(11)	Pb(26)-Pb(30)	3.2357(12)
Pb(9)-Au(1)	3.1706(11)	Pb(26)-Pb(31)	3.2520(12)
Pb(9)-Pb(10)	3.2031(14)	Pb(27)-Au(3)	3.0084(14)
Pb(9)-Au(7)	3.4466(12)	Pb(27)-Pb(28)	3.2331(16)
Pb(10)-Au(4)	3.0046(12)	Pb(27)-Pb(32)	3.2532(13)

Pb(10)-Au(1)	3.1813(11)	Pb(27)-Pb(31)	3.2573(14)
Pb(10)-Pb(11)	3.2282(12)	Pb(28)-Au(3)	3.0214(12)
Pb(11)-Au(4)	2.9441(12)	Pb(28)-Pb(33)	3.2481(13)
Pb(11)-Au(1)	3.0957(11)	Pb(28)-Pb(32)	3.2789(13)
Pb(12)-Au(2)	2.9876(12)	Pb(29)-Au(6)	2.9955(12)
Pb(12)-Pb(14)	3.1814(15)	Pb(29)-Au(3)	3.0993(13)
Pb(12)-Pb(13)	3.2319(13)	Pb(29)-Pb(30)	3.1977(11)
Pb(12)-Pb(17)	3.2380(14)	Pb(29)-Pb(33)	3.2269(12)
Pb(12)-Pb(15)	3.2433(14)	Pb(30)-Au(6)	3.0070(11)
Pb(12)-Pb(16)	3.2514(13)	Pb(30)-Au(3)	3.1571(11)
Pb(13)-Au(2)	3.0286(12)	Pb(30)-Pb(31)	3.2256(13)
Pb(13)-Pb(17)	3.1877(15)	Pb(31)-Au(6)	2.9472(13)
Pb(13)-Pb(14)	3.2225(15)	Pb(31)-Au(3)	3.1388(12)
Pb(13)-Pb(18)	3.2738(14)	Pb(31)-Pb(32)	3.2154(12)
Pb(13)-Pb(22)	3.3093(13)	Pb(32)-Au(6)	3.0266(13)
Pb(14)-Au(2)	3.0086(13)	Pb(32)-Au(3)	3.1652(12)
Pb(14)-Pb(15)	3.2324(14)	Pb(32)-Pb(33)	3.1937(14)
Pb(14)-Pb(18)	3.2807(14)	Pb(33)-Au(6)	3.0450(11)
Pb(14)-Pb(19)	3.2949(13)	Pb(33)-Au(3)	3.1413(11)
Pb(15)-Au(2)	2.9729(12)	Au(1)-Au(4)	2.7830(11)
Pb(15)-Pb(16)	3.1871(15)	Au(2)-Au(5)	2.7788(12)
Pb(15)-Pb(19)	3.2372(14)	Au(3)-Au(6)	2.7978(11)
Pb(15)-Pb(20)	3.3998(13)	Au(4)-Au(5)	2.7609(12)
Pb(16)-Au(2)	2.9950(12)	Au(4)-Au(7)	2.8860(11)
Pb(16)-Pb(17)	3.1989(14)	Au(4)-Au(8)	2.9040(11)
Pb(16)-Pb(20)	3.4784(14)	Au(5)-Au(8)	2.9740(12)
Pb(17)-Au(2)	3.0106(13)	Au(5)-Au(7)	2.9897(11)
Pb(17)-Pb(21)	3.2704(13)	Au(6)-Au(8)	2.8417(12)
Pb(17)-Pb(22)	3.2964(13)	Au(6)-Au(7)	2.8461(12)
Pb(18)-Au(5)	2.9746(11)	Au(7)-Au(8)	3.3592(12)
Pb(18)-Au(2)	3.1188(12)		

Supplementary Table 4. Selected interatomic distances (in Å) in the X-ray geometry of compound 3.

	X-ray		X-ray
Au(3)-Au(7)	2.803(2)	Pb(21)-Pb(20)	3.240(2)
Au(5)-Au(10)	2.9251(19)	Pb(21)-Pb(22)	3.1675(19)
Au(5)-Au(1)	2.821(2)	Pb(21)-Pb(16)	3.2979(19)
Au(6)-Au(2)	2.811(2)	Pb(21)-Pb(15)	3.256(2)
Au(8)-Au(4)	2.816(2)	Pb(20)-Pb(15)	3.297(2)
Au(9)-Au(6)	2.902(2)	Pb(20)-Pb(19)	3.173(2)
Au(9)-Au(8)	2.914(2)	Pb(20)-Pb(14)	3.279(2)
Au(9)-Au(7)	2.921(2)	Pb(22)-Pb(16)	3.228(2)
Au(10)-Au(8)	2.932(2)	Pb(22)-Pb(17)	3.271(2)
Au(10)-Au(7)	2.909(2)	Pb(22)-Pb(18)	3.258(2)
Au(11)-Au(5)	2.943(2)	Pb(22)-Pb(42)	3.635(2)
Au(11)-Au(6)	2.9141(19)	Pb(10)-Pb(11)	3.149(2)
Au(11)-Au(8)	2.9137(19)	Pb(10)-Pb(9)	3.261(2)
Au(12)-Au(5)	2.8941(19)	Pb(10)-Pb(6)	3.280(2)
Au(12)-Au(6)	2.942(2)	Pb(10)-Pb(5)	3.258(2)
Au(12)-Au(7)	2.919(2)	Pb(16)-Pb(15)	3.230(2)
Au(1)-Pb(10)	3.136(2)	Pb(16)-Pb(17)	3.255(2)
Au(1)-Pb(8)	3.132(2)	Pb(16)-Pb(12)	3.221(2)
Au(1)-Pb(11)	3.138(2)	Pb(15)-Pb(12)	3.279(2)
Au(1)-Pb(7)	3.173(2)	Pb(15)-Pb(14)	3.198(2)
Au(1)-Pb(9)	3.160(2)	Pb(8)-Pb(7)	3.200(3)
Au(1)-Pb(6)	3.020(2)	Pb(8)-Pb(9)	3.185(3)
Au(1)-Pb(3)	3.010(2)	Pb(8)-Pb(3)	3.270(2)
Au(1)-Pb(1)	3.000(2)	Pb(8)-Pb(4)	3.299(2)
Au(1)-Pb(2)	3.003(2)	Pb(17)-Pb(18)	3.280(2)
Au(1)-Pb(5)	2.984(3)	Pb(17)-Pb(12)	3.247(2)
Au(1)-Pb(4)	3.010(2)	Pb(17)-Pb(13)	3.215(2)
Au(2)-Pb(20)	3.1702(18)	Pb(11)-Pb(7)	3.228(2)
Au(2)-Pb(22)	3.111(2)	Pb(11)-Pb(6)	3.272(2)
Au(2)-Pb(16)	3.018(2)	Pb(11)-Pb(2)	3.278(2)
Au(2)-Pb(15)	3.021(2)	Pb(30)-Pb(31)	3.217(2)
Au(2)-Pb(17)	3.0050(19)	Pb(30)-Pb(29)	3.229(2)
Au(2)-Pb(18)	3.144(2)	Pb(30)-Pb(25)	3.276(2)
Au(2)-Pb(19)	3.122(2)	Pb(30)-Pb(24)	3.297(3)
Au(2)-Pb(12)	2.969(2)	Pb(18)-Pb(19)	3.183(2)
Au(2)-Pb(14)	2.987(2)	Pb(18)-Pb(13)	3.303(2)
Au(2)-Pb(13)	3.020(2)	Pb(7)-Pb(3)	3.320(2)
Au(2)-Pb(21)	3.174(2)	Pb(7)-Pb(2)	3.295(2)
Au(3)-Pb(30)	3.142(2)	Pb(7)-Pb(33)	3.674(2)
Au(3)-Pb(31)	3.148(2)	Pb(19)-Pb(14)	3.264(2)

Au(3)-Pb(29)	3.141(2)	Pb(19)-Pb(13)	3.274(2)
Au(3)-Pb(25)	3.003(2)	Pb(12)-Pb(14)	3.230(2)
Au(3)-Pb(23)	2.981(2)	Pb(12)-Pb(13)	3.246(3)
Au(3)-Pb(26)	3.023(2)	Pb(14)-Pb(13)	3.234(2)
Au(3)-Pb(33)	3.145(2)	Pb(9)-Pb(5)	3.302(2)
Au(3)-Pb(24)	3.006(3)	Pb(9)-Pb(4)	3.273(2)
Au(3)-Pb(27)	2.999(2)	Pb(31)-Pb(25)	3.272(3)
Au(3)-Pb(32)	3.164(3)	Pb(31)-Pb(26)	3.290(2)
Au(3)-Pb(28)	3.026(2)	Pb(31)-Pb(32)	3.171(3)
Au(4)-Pb(44)	3.131(2)	Pb(29)-Pb(33)	3.167(3)
Au(4)-Pb(42)	3.160(2)	Pb(29)-Pb(24)	3.258(3)
Au(4)-Pb(34)	2.998(2)	Pb(29)-Pb(28)	3.256(3)
Au(4)-Pb(40)	3.154(3)	Pb(44)-Pb(40)	3.246(3)
Au(4)-Pb(43)	3.155(2)	Pb(44)-Pb(43)	3.204(3)
Au(4)-Pb(35)	2.983(2)	Pb(44)-Pb(35)	3.340(2)
Au(4)-Pb(36)	3.013(2)	Pb(44)-Pb(39)	3.284(3)
Au(4)-Pb(39)	2.999(2)	Pb(6)-Pb(1)	3.251(3)
Au(4)-Pb(41)	3.158(2)	Pb(6)-Pb(2)	3.223(3)
Au(4)-Pb(38)	3.007(3)	Pb(6)-Pb(5)	3.191(3)
Au(4)-Pb(37)	2.983(2)	Pb(25)-Pb(23)	3.262(3)
Au(5)-Pb(10)	2.980(2)	Pb(25)-Pb(26)	3.222(3)
Au(5)-Pb(8)	2.986(2)	Pb(25)-Pb(24)	3.218(3)
Au(5)-Pb(11)	3.015(2)	Pb(42)-Pb(43)	3.179(3)
Au(5)-Pb(7)	3.019(2)	Pb(42)-Pb(41)	3.250(3)
Au(5)-Pb(9)	2.988(2)	Pb(42)-Pb(38)	3.323(3)
Au(6)-Pb(21)	3.018(2)	Pb(42)-Pb(37)	3.257(3)
Au(6)-Pb(20)	2.9647(19)	Pb(34)-Pb(35)	3.215(3)
Au(6)-Pb(22)	3.020(2)	Pb(34)-Pb(36)	3.244(3)
Au(6)-Pb(18)	2.993(2)	Pb(34)-Pb(39)	3.216(3)
Au(6)-Pb(19)	2.984(2)	Pb(34)-Pb(38)	3.233(3)
Au(7)-Pb(30)	2.959(2)	Pb(34)-Pb(37)	3.269(3)
Au(7)-Pb(31)	3.011(2)	Pb(40)-Pb(35)	3.272(3)
Au(7)-Pb(29)	3.014(2)	Pb(40)-Pb(36)	3.287(3)
Au(7)-Pb(33)	2.977(2)	Pb(40)-Pb(41)	3.176(3)
Au(7)-Pb(32)	3.018(2)	Pb(3)-Pb(1)	3.243(3)
Au(8)-Pb(44)	2.964(2)	Pb(3)-Pb(2)	3.224(3)
Au(8)-Pb(42)	2.993(2)	Pb(3)-Pb(4)	3.224(3)
Au(8)-Pb(40)	3.014(2)	Pb(23)-Pb(26)	3.229(3)
Au(8)-Pb(43)	3.030(2)	Pb(23)-Pb(24)	3.241(3)
Au(8)-Pb(41)	3.006(2)	Pb(23)-Pb(27)	3.260(3)
Au(12)-Pb(11)	3.5298(19)	Pb(23)-Pb(28)	3.246(2)
Au(12)-Pb(29)	3.516(2)	Pb(1)-Pb(2)	3.245(3)
Au(9)-Pb(22)	3.525(2)	Pb(1)-Pb(5)	3.266(3)

Au(9)-Pb(31)	3.534(2)	Pb(1)-Pb(4)	3.255(3)
Au(9)-Pb(41)	3.484(2)	Pb(43)-Pb(39)	3.326(2)
Au(10)-Pb(8)	3.474(2)	Pb(43)-Pb(38)	3.255(3)
Pb(33)-Pb(28)	3.282(3)	Pb(35)-Pb(36)	3.186(3)
Pb(39)-Pb(38)	3.211(3)	Pb(35)-Pb(39)	3.192(3)
Pb(5)-Pb(4)	3.223(3)	Pb(26)-Pb(27)	3.215(3)
Pb(24)-Pb(28)	3.242(3)	Pb(26)-Pb(32)	3.306(2)
Pb(41)-Pb(37)	3.275(3)	Pb(36)-Pb(41)	3.268(2)
Pb(27)-Pb(32)	3.277(3)	Pb(36)-Pb(37)	3.212(3)
Pb(27)-Pb(28)	3.227(3)	Pb(33)-Pb(27)	3.291(2)
Pb(38)-Pb(37)	3.207(4)	Pb(33)-Pb(32)	3.260(3)

.

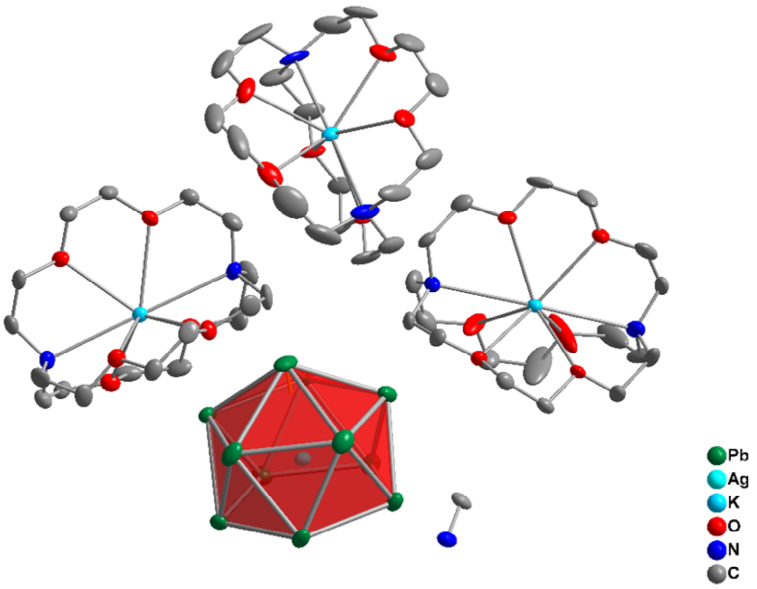

Supplementary Figure 2. **Asymmetric unit of compound 1 with the cluster fragment.** Thermal ellipsoids are drawn at 50% probability. The minor components are omitted for clarity.

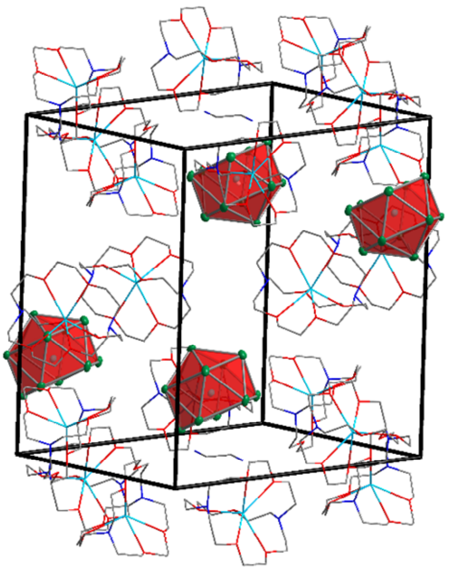

Supplementary Figure 3. Unit cell of compound 1. Minor components in the cluster site are omitted for clarity.

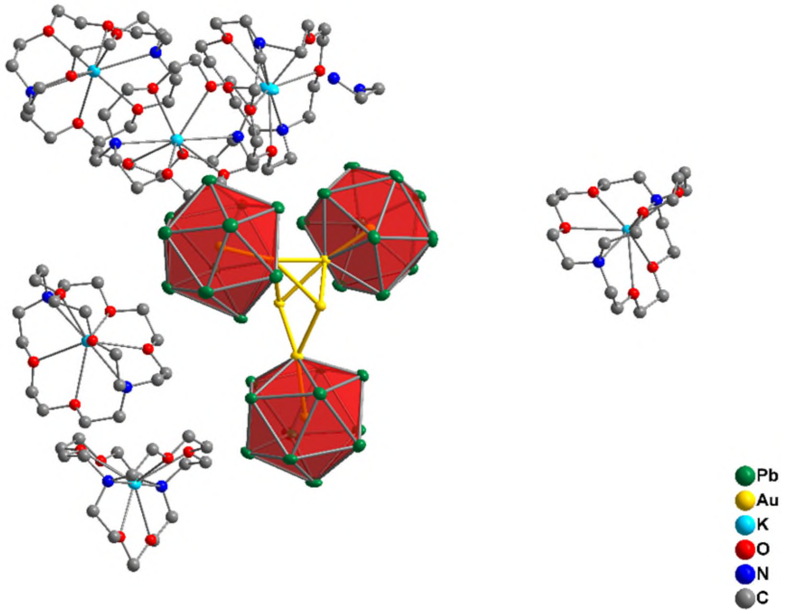

Supplementary Figure 4. Asymmetric unit of compound 2 with the cluster fragment. Thermal ellipsoids are drawn at 50% probability. The minor components are omitted for clarity.

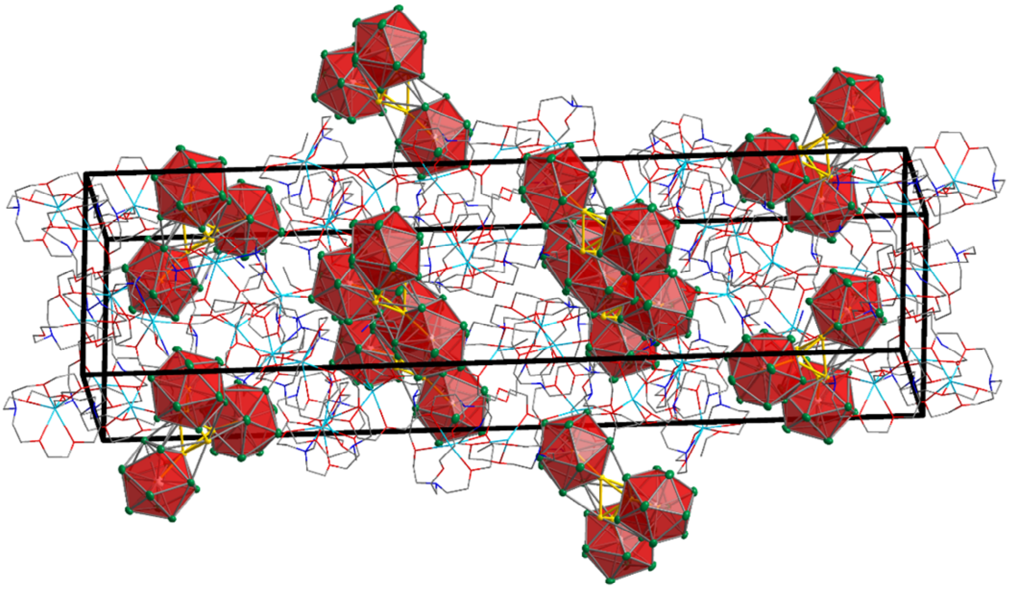

Supplementary Figure 5. Unit cell of compound 2. Minor component in the cluster site are omitted for clarity.

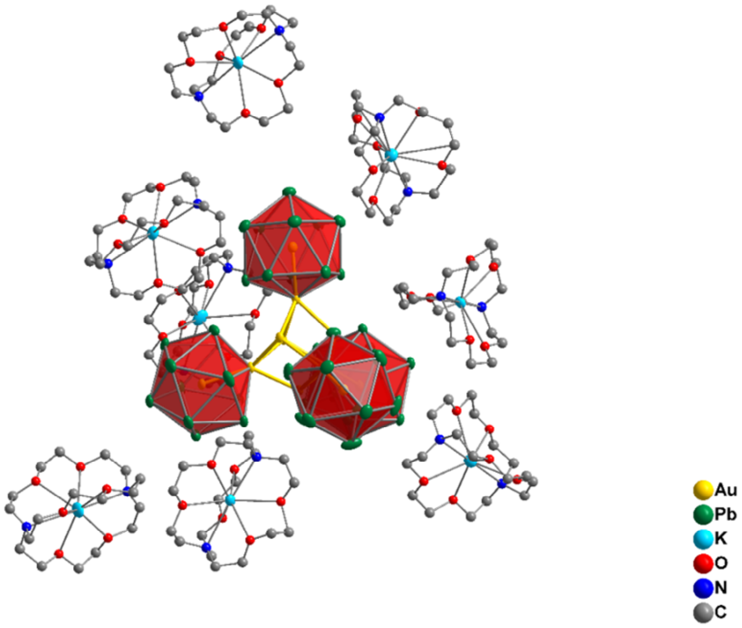

Supplementary Figure 6. Asymmetric unit of compound 3 with the cluster fragment. Thermal ellipsoids are drawn at 50% probability. The minor components are omitted for clarity.

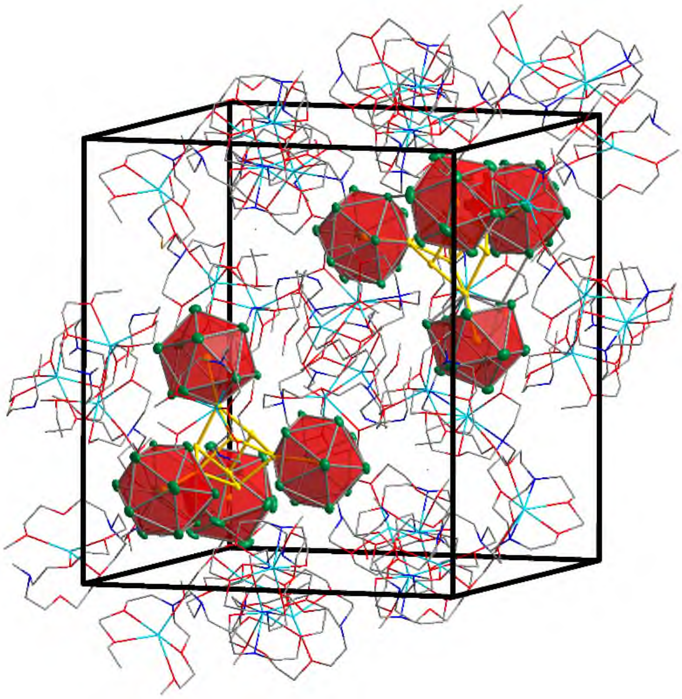

Supplementary Figure 7. Unit cell of compound 3. Minor component in the cluster site are omitted for clarity.

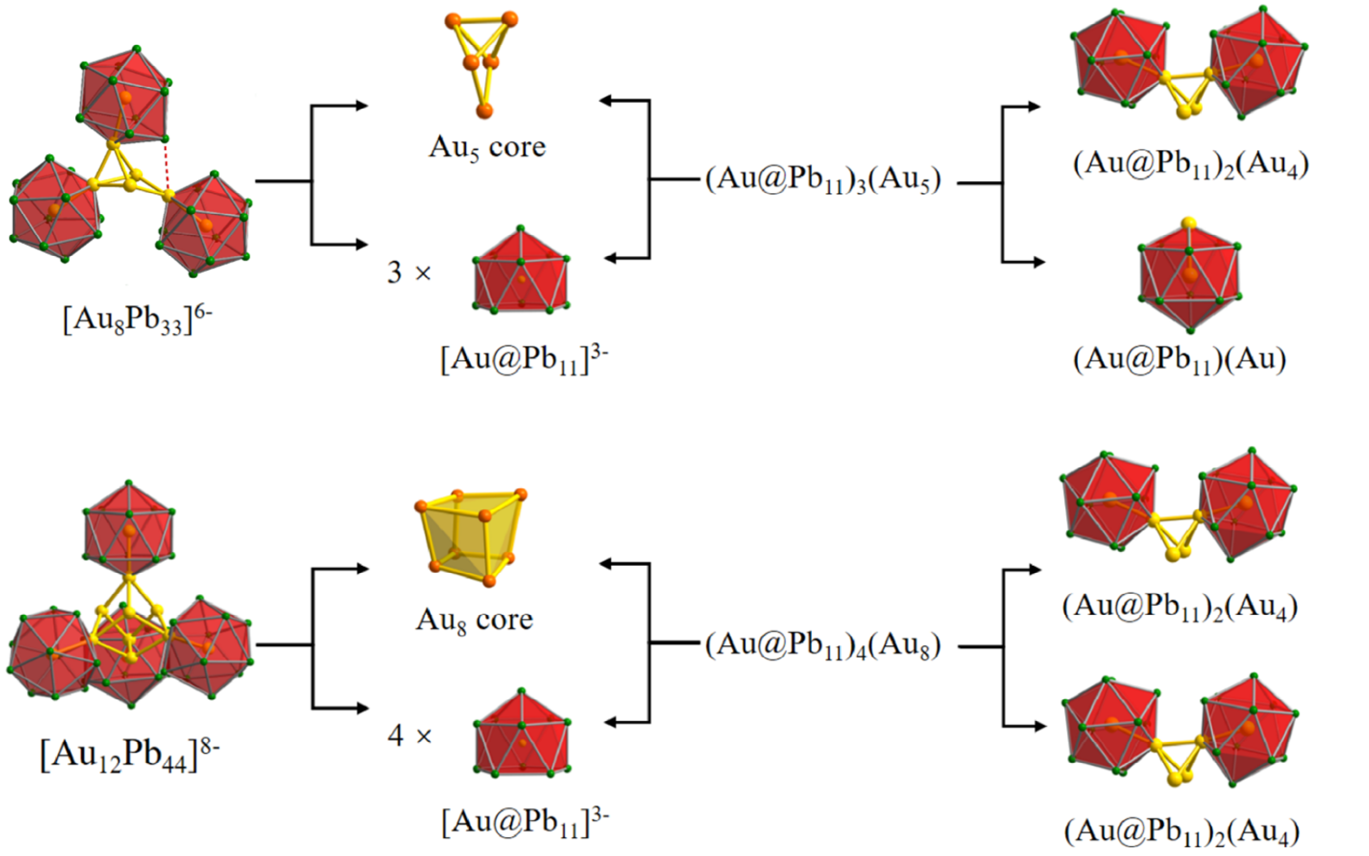

Supplementary Figure 8. The decomposition and recombination of $[Au_8Pb_{33}]^{6-}$ and $[Au_{12}Pb_{44}]^{8-}$ clusters.

Supplementary Table 5. Summary of structural data for $[M@Pb_{11}]$ units (M = Ag, Au).

		Pb-M distances			Pb-Pb distances		
	$[M@Pb_{11}]$ unit	average(Å)	range(Å)	Variance[a] ($\sigma^2 \times 10^{-3}$)	average(Å)	range(Å)	Variance[a] ($\sigma^2 \times 10^{-3}$)
$[Ag@Pb_{11}]^{3-}$		3.052	0.11	1.96	3.208	0.16	2.36
$[Au_8Pb_{33}]^{6-}$	I[b]	3.043	0.246	10.6	3.245	0.132	1.33
	II[b]	3.055	0.386	14.6	3.257	0.297	4.74
	III[b]	3.048	0.218	7.32	3.242	0.087	0.509
$[Au_{12}Pb_{44}]^{8-}$	IV[c]	3.070	0.189	5.80	3.249	0.171	1.64
	V[c]	3.067	0.205	5.96	3.245	0.136	1.43
	VI[c]	3.070	0.183	5.65	3.248	0.139	1.28
	VII[c]	3.067	0.177	6.64	3.245	0.164	2.10

[a] Variance is defined as: $\sigma^2 = [\sum_{i=1}^{N}(x_i - \bar{x})^2]/(N-1)$. [b] $[Au_8Pb_{33}]^{6-}$ can be divided into an Au_5 core and three $[Au@Pb_{11}]$ units, I, II and III. The capped Pb atoms of units I, II and III for $[Au_8Pb_{33}]^{6-}$ are Pb1, Pb12 and Pb23. [c] $[Au_{12}Pb_{44}]^{6-}$ can be divided into an Au_8 core and four $[Au@Pb_{11}]$ units, IV, V, VI and VII. The capped Pb atoms of units IV, V, VI and VII for $[Au_{12}Pb_{44}]^{8-}$ are Pb1, Pb12, Pb23 and Pb34.

Section 3: Electrospray Ionization Mass Spectrometry (ESI-MS) Analysis

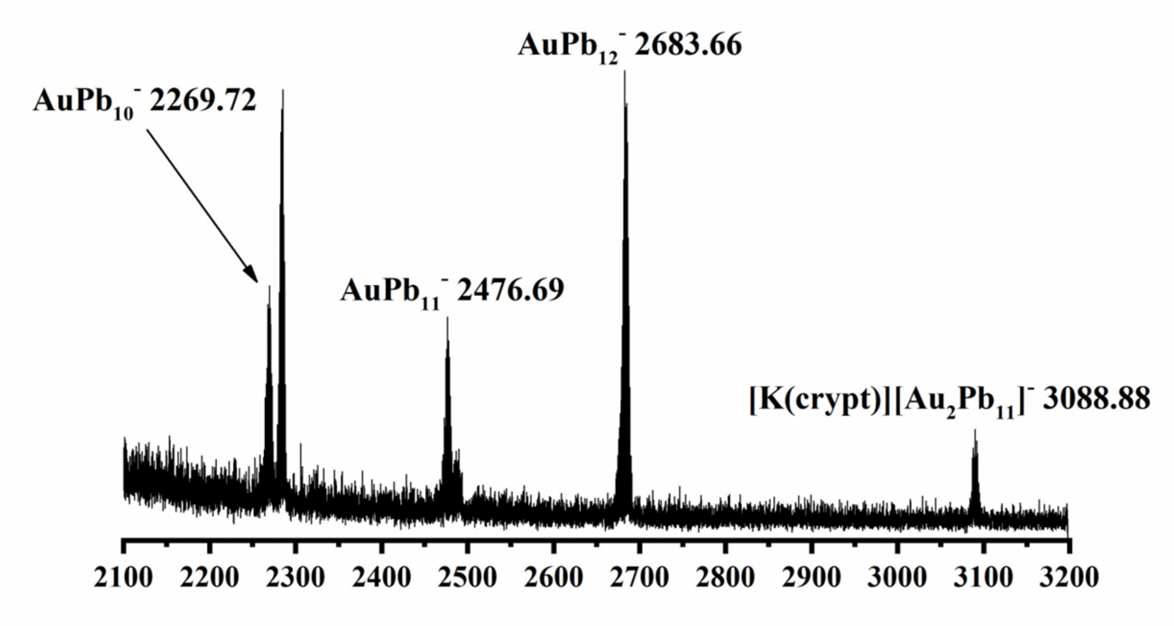

Supplementary Figure 9. ESI-MS of the reaction of K_4Pb_9 with $Au(Mes)PPh_3$ in negative-ion mode.

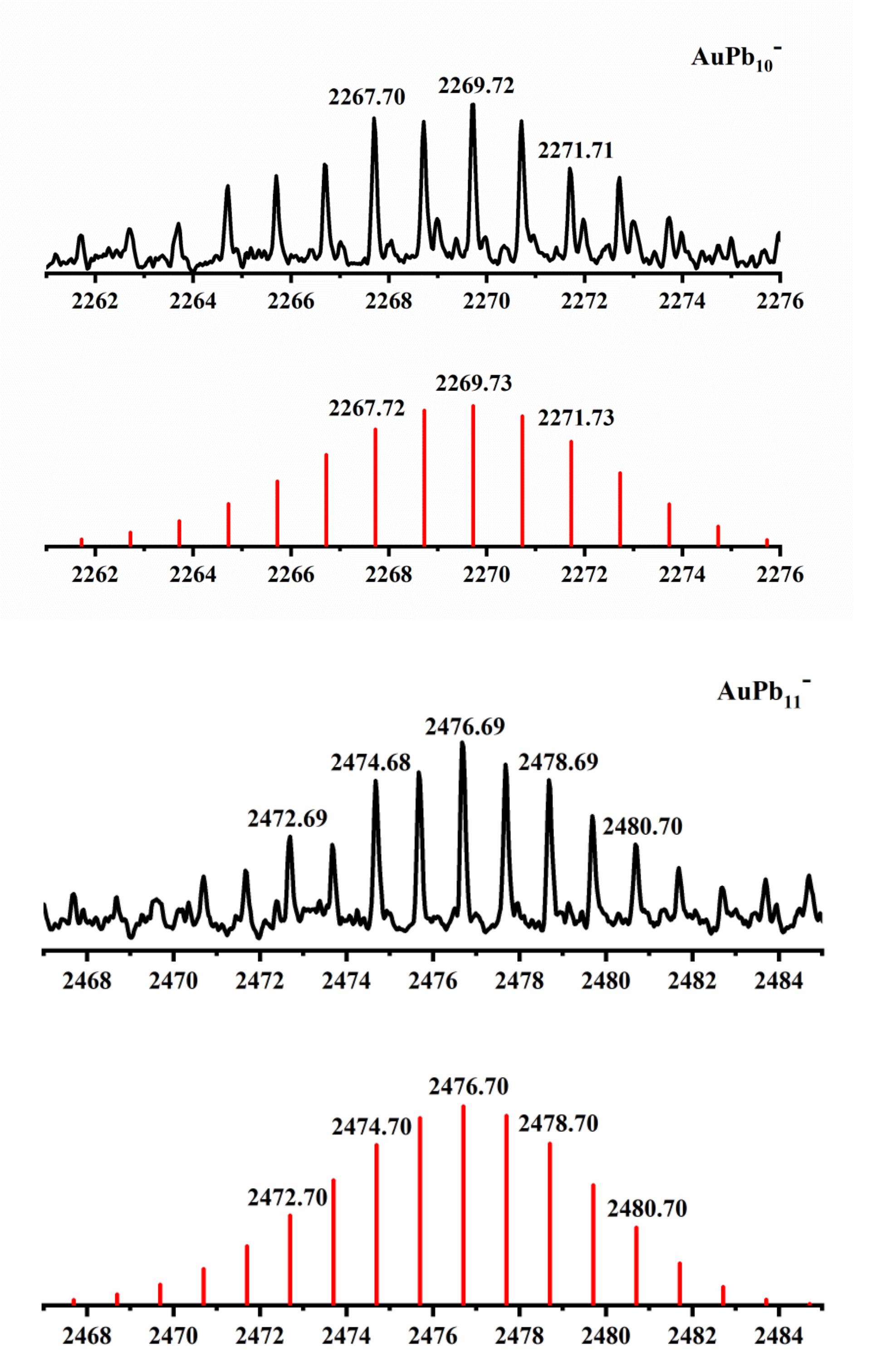

Supplementary Figure 10. Experimental (black) and simulated (red) spectra of the fragments $[AuPb_{10}]^-$ and $[AuPb_{11}]^-$.

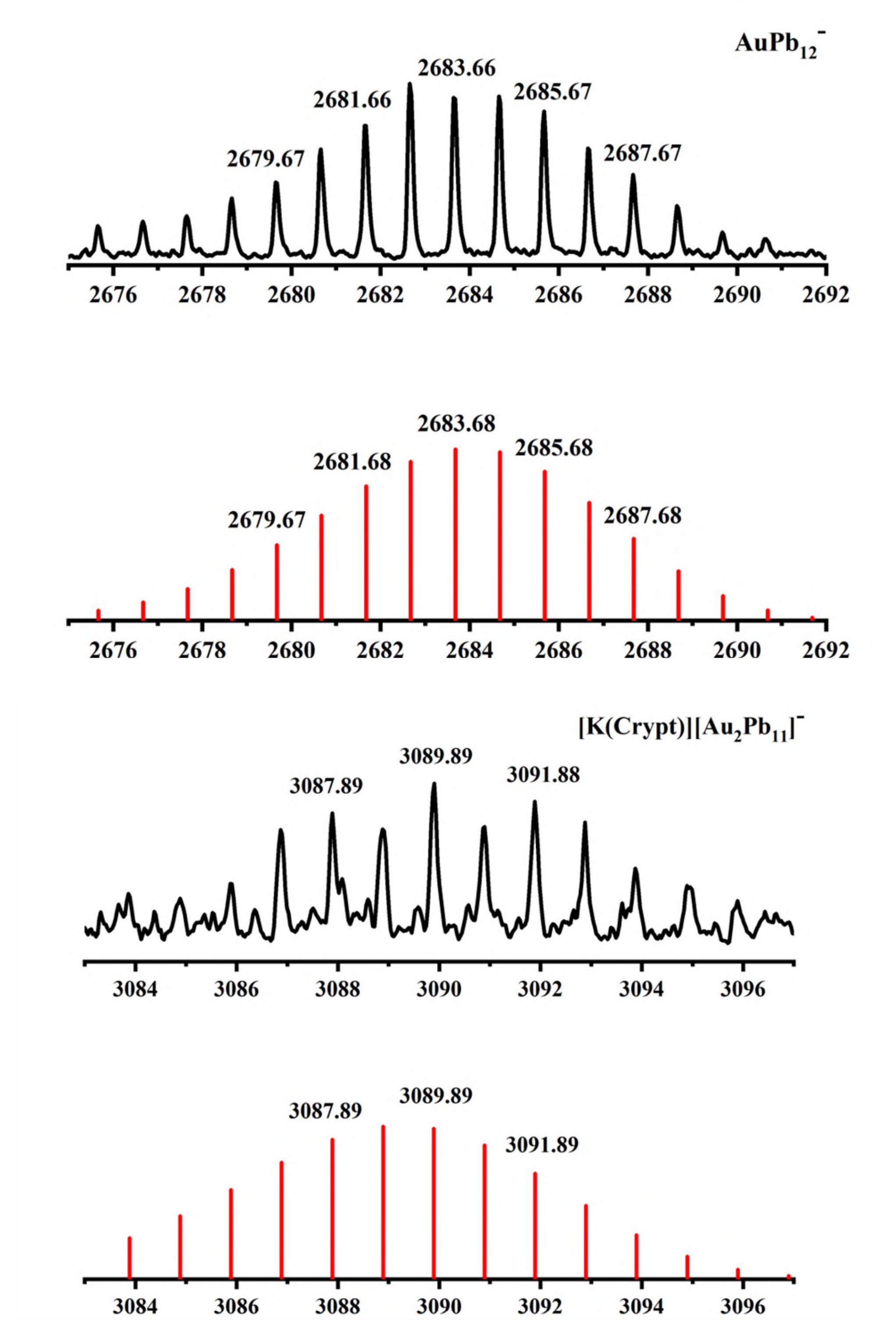

Supplementary Figure 11. Experimental (black) and simulated (red) spectra of the fragments $[AuPb_{12}]^{-}$ and $[K([2.2.2]crypt)][Au_2Pb_{11}]^{-}$.

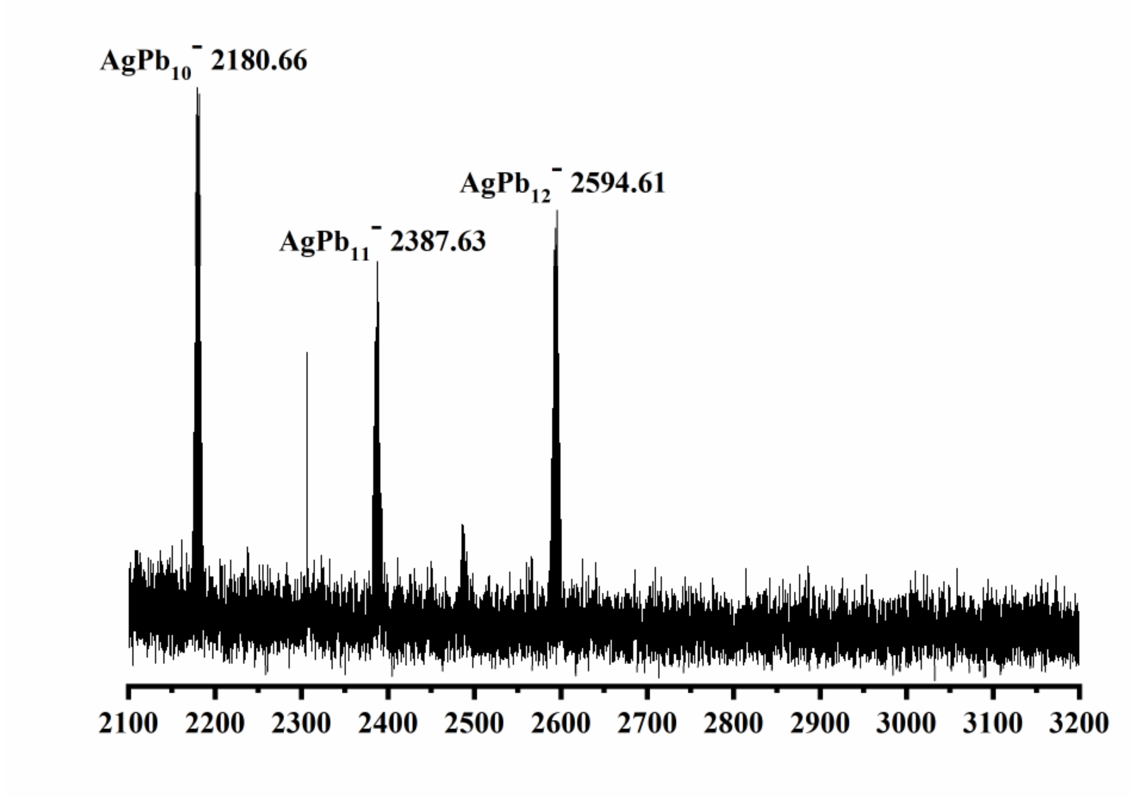

Supplementary Figure 12. ESI-MS of the reaction of K_4Pb_9 with $(AgMes)_4$ in negative-ion mode.

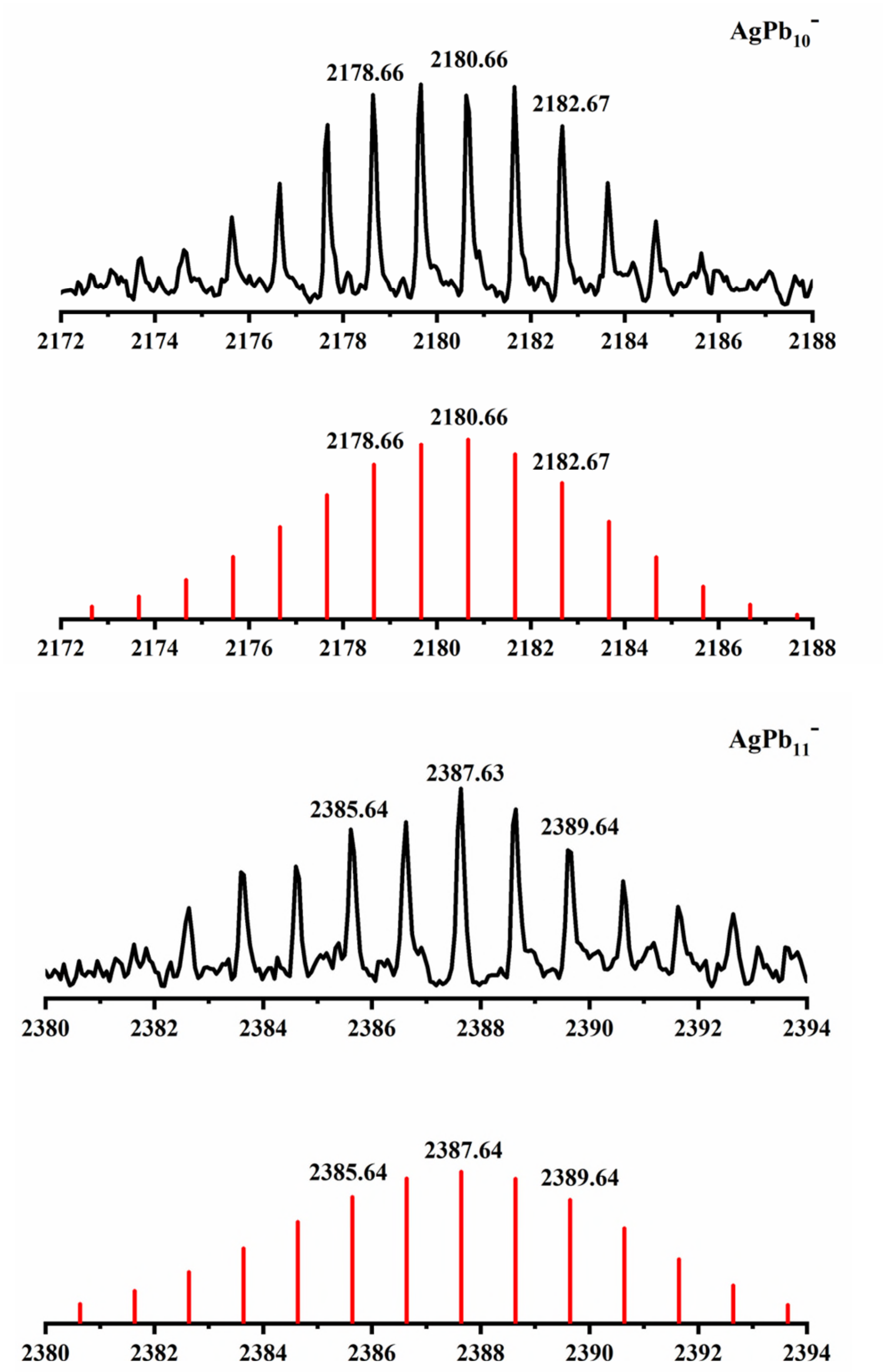

Supplementary Figure 13. Experimental (black) and simulated (red) spectra of the fragments $[AgPb_{10}]^-$ and $[AgPb_{11}]^-$.

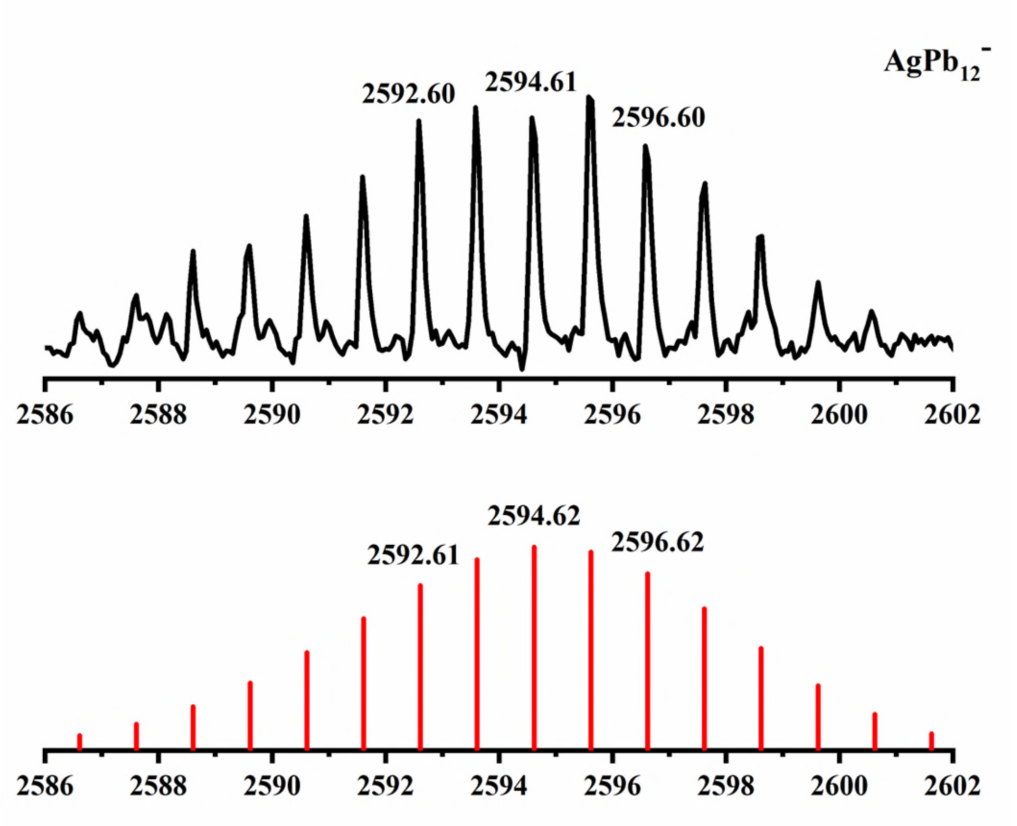

Supplementary Figure 14. Experimental (black) and simulated (red) spectra of the fragment $[AgPb_{12}]^-$.

Section 4: Energy Dispersive X-ray (EDX) Spectroscopic Analysis

1

2

3

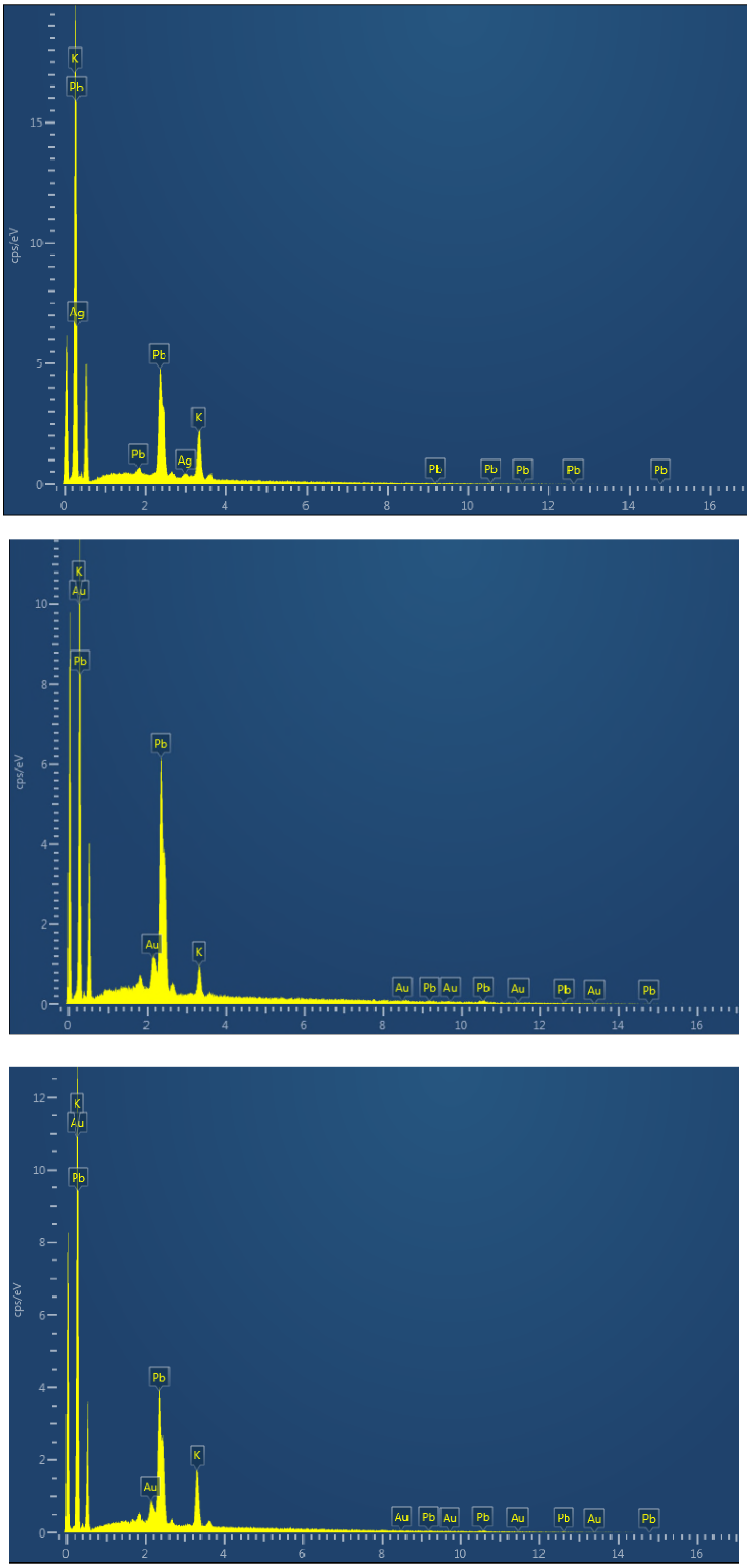

Supplementary Figure 15. EDX analysis of complex 1 (K, Ag, Pb), 2 and 3 (K, Au, Pb).

Section 5: Quantum chemical studies

Supplementary Table 6. Optimized structures and total energies of the M^+ ions and the fragments $[M@Pb_{11}]^{3-}$, and $[M@MPb_{11}]^{2-}$ (M = Ag, Au). Note total energies are reported relative to the sum of the atomic fragments.

$[Ag@Pb_{11}]^{3-}$ E = -46.87636878 eV

Ag	0.000000	0.000000	0.047099
Pb	-2.257975	1.640515	1.423098
Pb	0.862470	2.654409	1.423098
Pb	-0.888120	-2.733352	-1.303447
Pb	0.862470	-2.654409	1.423098
Pb	-2.257975	-1.640515	1.423098
Pb	2.791011	0.000000	1.423098
Pb	2.325128	1.689304	-1.303447
Pb	-0.888120	2.733352	-1.303447
Pb	0.000000	0.000000	-3.035305
Pb	-2.874016	0.000000	-1.303447
Pb	2.325128	-1.689304	-1.303447

***exo*-$[AgPb_{11}]^{3-}$ E = --46.67434765 eV**

Ag	0.000000	0.000000	2.027539
Pb	-2.317472	1.683742	1.470320
Pb	0.885196	2.724352	1.470320
Pb	-0.866653	-2.667283	-1.238208
Pb	0.885196	-2.724352	1.470320
Pb	-2.317472	-1.683742	1.470320
Pb	2.864553	0.000000	1.470320
Pb	2.268927	1.648472	-1.238208
Pb	-0.866653	2.667283	-1.238208
Pb	0.000000	0.000000	-2.949836
Pb	-2.804548	0.000000	-1.238208
Pb	2.268927	-1.648472	-1.238208

$[Ag@AgPb_{11}]^{2-}$ E = -46.93058598 eV

Ag	0.000000	0.000000	-0.189660
Ag	0.000000	0.000000	2.540162
Pb	-2.298961	1.670293	1.470920
Pb	0.878125	2.702590	1.470920
Pb	-0.884375	-2.721827	-1.299856
Pb	0.878125	-2.702590	1.470920
Pb	-2.298961	-1.670293	1.470920
Pb	2.841672	0.000000	1.470920
Pb	2.315324	1.682181	-1.299856
Pb	-0.884375	2.721827	-1.299856
Pb	0.000000	0.000000	-3.132634
Pb	-2.861898	0.000000	-1.299856

Pb	2.315324	-1.682181	-1.299856

$[Au@Pb_{11}]^{3-}$ E = -47.59583229 eV

Au	0.000000	0.000000	0.044112
Pb	-2.254460	1.637961	1.432745
Pb	0.861127	2.650277	1.432745
Pb	-0.887227	-2.730603	-1.294484
Pb	0.861127	-2.650277	1.432745
Pb	-2.254460	-1.637961	1.432745
Pb	2.786666	0.000000	1.432745
Pb	2.322790	1.687605	-1.294484
Pb	-0.887227	2.730603	-1.294484
Pb	0.000000	0.000000	-3.024368
Pb	-2.871126	0.000000	-1.294484
Pb	2.322790	-1.687605	-1.294484

***exo*-$[AuPb_{11}]^{3-}$ E = -47.57550738 eV**

Au	0.000000	0.000000	2.062429
Pb	-2.316805	1.683257	1.507013
Pb	0.884941	2.723568	1.507013
Pb	-0.868141	-2.671862	-1.205630
Pb	0.884941	-2.723568	1.507013
Pb	-2.316805	-1.683257	1.507013
Pb	2.863729	0.000000	1.507013
Pb	2.272821	1.651301	-1.205630
Pb	-0.868141	2.671862	-1.205630
Pb	0.000000	0.000000	-2.910267
Pb	-2.809362	0.000000	-1.205630
Pb	2.272821	-1.651301	-1.205630

$[Au@AuPb_{11}]^{3-}$ E = -48.38097763 eV

Au	0.000000	0.000000	-0.327418
Au	0.000000	0.000000	2.429310
Pb	-2.308357	1.677120	1.508460
Pb	0.881714	2.713636	1.508460
Pb	-0.888106	-2.733311	-1.267659
Pb	0.881714	-2.713636	1.508460
Pb	-2.308357	-1.677120	1.508460
Pb	2.853286	0.000000	1.508460
Pb	2.325093	1.689279	-1.267659
Pb	-0.888106	2.733311	-1.267659
Pb	0.000000	0.000000	-3.194624
Pb	-2.873973	0.000000	-1.267659
Pb	2.325093	-1.689279	-1.267659

Energy decomposition analysis (EDA) for the interaction between M^+ and $[M@Pb_{11}]^{3-}$, M = Ag and Au.

Supplementary Table 7. Total interaction energies and their components (in eV) for the fragmentation of $[M@MPb_{11}]^{2-}$ into $[M@Pb_{11}]^{3-}$ + M^+.

		M = Au	M = Ag
$\mathbf{E_{steric}}$		–9.32	–9.12
$\mathbf{E_{orb}}$			
	$\mathbf{a_1}$	–4.58	–2.87
	$\mathbf{a_2}$	0.0	0.0
	$\mathbf{e_1}$	–2.16	-1.85
	$\mathbf{e_2}$	–0.76	–0.50
	**total**	–7.50	–5.22
$\mathbf{E_{solv}}$		–5.22	–5.10
$\mathbf{E_{tot}}$		–21.95	–19.36

Supplementary Discussion

D_{2d}-symmetrized coordinates of $[Au_{12}Pb_{44}]^{8-}$ (not optimized at the DFT level)

In order to simplify the analysis of the electronic structure, we have imposed D_{2d} symmetry on the $[Au_{12}Pb_{44}]^{8-}$ cluster. This amounts to applying rotations of the Pb_{11} units about the 5-fold rotational axes containing both Au atoms: the Au_{12} core is almost unaltered. The major effect of this symmetrization is on the secondary Au…Pb distances discussed in the text. The histogram in Figure S16 compares the distribution of these distances in the crystal structures (blue) and the symmetrized structure (red). The key feature is that the mean distance is almost the same in the two distributions, confirming that the nature of the bonding has not been changed in any fundamental way by the imposition of higher symmetry.

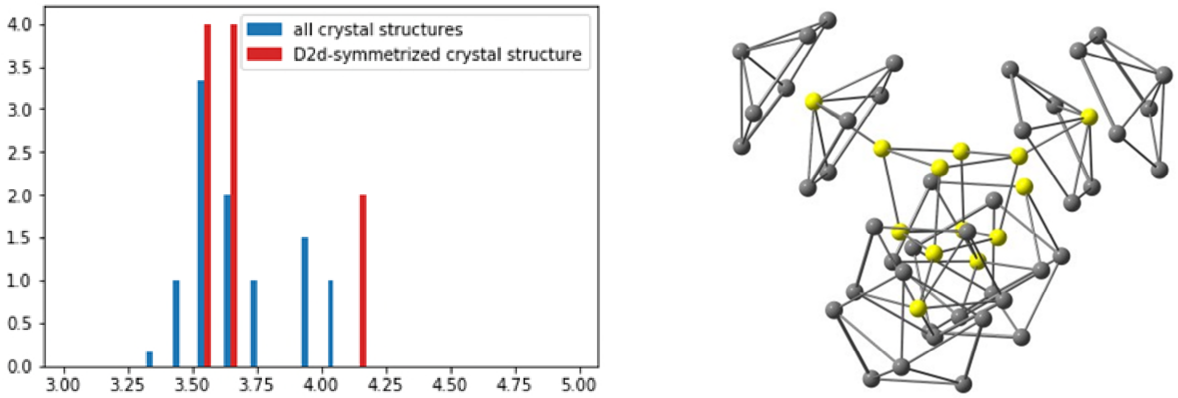

Supplementary Figure 16**.** Secondary Au…Pb distances in the crystal structures (blue) and the D_{2d}-symmetrized structure (red).

D_{2d}-symmetrized Coordinates:

Au	-1.197969059	-1.197969059	-1.288098630
Au	-1.197969059	1.197969059	1.288098630
Au	1.197969059	-1.197969059	1.288098630
Au	1.197969059	1.197969059	-1.288098630
Au	-1.675890536	1.675890536	-1.538848817
Au	-1.675890536	-1.675890536	1.538848817
Au	1.675890536	-1.675890536	-1.538848817
Au	1.675890536	1.675890536	1.538848817
Au	-3.368541190	-3.368541190	3.012744528
Au	-3.368541190	3.368541190	-3.012744528
Au	3.368541190	3.368541190	3.012744528
Au	3.368541190	-3.368541190	-3.012744528
Pb	-2.091775464	-4.362444749	0.327635790

Pb	-4.362444749	-2.091775464	0.327635790
Pb	-0.315484800	-3.956896564	2.884639316
Pb	-3.956896564	0.315484800	-2.884639316
Pb	-2.618249549	-6.257926523	2.953410191
Pb	-5.107499688	-5.107499688	1.340012782
Pb	-0.315484800	3.956896564	-2.884639316
Pb	-2.182688617	-4.425135046	5.523298158
Pb	-4.362444749	2.091775464	-0.327635790
Pb	1.412997592	1.412997592	4.467648542
Pb	-1.412997592	-1.412997592	4.467648542
Pb	-2.091775464	4.362444749	-0.327635790
Pb	-3.956896564	-0.315484800	2.884639316
Pb	-5.166343149	-5.166343149	4.576237170
Pb	-6.257926523	-2.618249549	2.953410191
Pb	-1.412997592	1.412997592	-4.467648542
Pb	-4.425135046	-2.182688617	5.523298158
Pb	3.956896564	0.315484800	2.884639316
Pb	0.315484800	3.956896564	2.884639316
Pb	1.412997592	-1.412997592	-4.467648542
Pb	-6.257926523	2.618249549	-2.953410191
Pb	4.425135046	2.182688617	5.523298158
Pb	2.091775464	-4.362444749	-0.327635790
Pb	5.166343149	-5.166343149	-4.576237170
Pb	3.956896564	-0.315484800	-2.884639316
Pb	-2.618249549	6.257926523	-2.953410191
Pb	5.166343149	5.166343149	4.576237170
Pb	-5.166343149	5.166343149	-4.576237170
Pb	-5.107499688	5.107499688	-1.340012782
Pb	0.315484800	-3.956896564	-2.884639316
Pb	4.425135046	-2.182688617	-5.523298158
Pb	6.257926523	2.618249549	2.953410191
Pb	6.257926523	-2.618249549	-2.953410191
Pb	2.091775464	4.362444749	0.327635790
Pb	2.182688617	-4.425135046	-5.523298158
Pb	-4.425135046	2.182688617	-5.523298158
Pb	-2.182688617	4.425135046	-5.523298158
Pb	2.182688617	4.425135046	5.523298158
Pb	4.362444749	-2.091775464	-0.327635790
Pb	5.107499688	5.107499688	1.340012782
Pb	4.362444749	2.091775464	0.327635790
Pb	2.618249549	6.257926523	2.953410191
Pb	2.618249549	-6.257926523	-2.953410191
Pb	5.107499688	-5.107499688	-1.340012782

Total energies for the D_{2d}-symmetrised geometry:
$^3A_2$: –203.6907 eV
^3E: –203.6904 eV
$^1A_1$: –203.6569 eV

Structures and energies of D_{2d}-symmetric structure with permuted Au and Pb atoms ($^1A_1$ state)

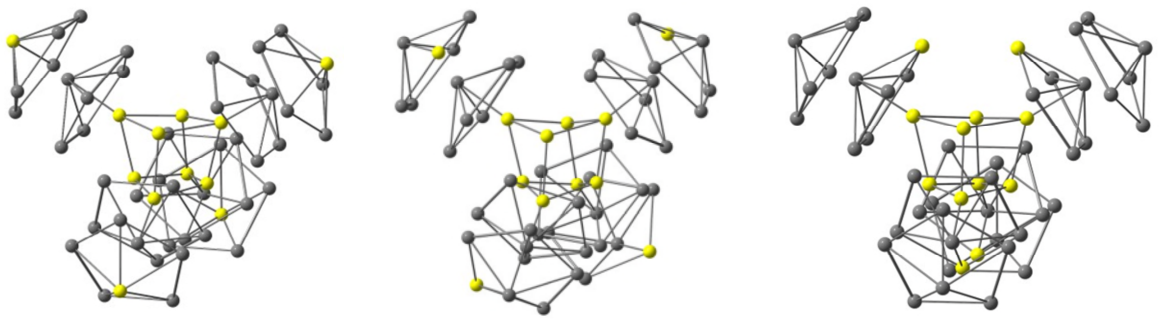

E = –190.47 eV **E = –188.72 eV** **E = –187.04 eV**

Permutations of the surface Au centers

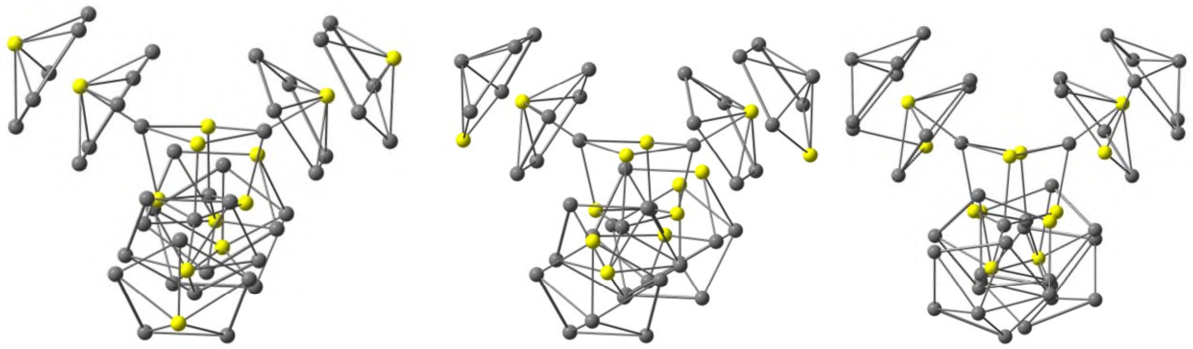

E = –194.35 eV **E = –194.74 eV** **E = –196.46 eV**

Permutations of the capping Au centers

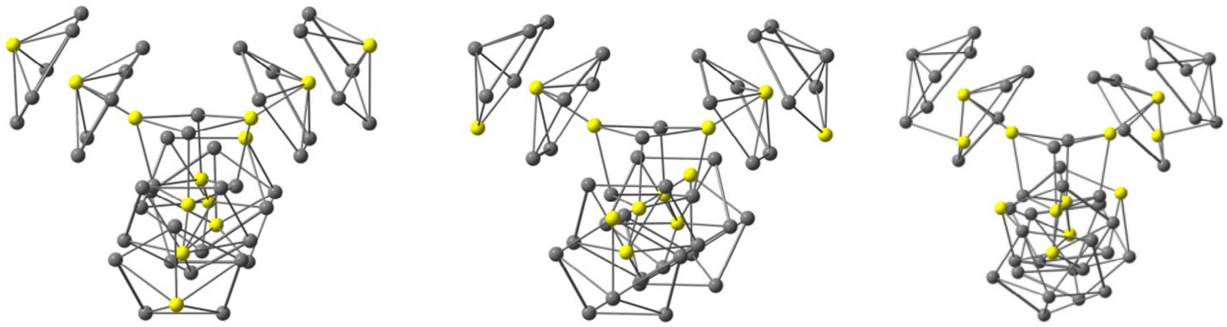

E = –202.92 eV **E = –202.48 eV** **E = –202.88 eV**

Supplementary Figure 17. Total energies of structures with Au and Pb permuted into different positions.

Fragment analysis of the interaction between $[Au_8]^{4+}$ and $([Au@Pb_{11}]^{3-})_4$.

The analysis below relates to the $^1A_1$ state of $[Au_{12}Pb_{44}]^{8-}$. The Au_4 fragment is also prepared in the singlet configuration. This fragment analysis was performed using the D_{2d} symmetry of the cluster, rather than the idealized T_d symmetry presented in Figure 7. The Figure is reproduced below, with the real D_{2d}-symmetric orbitals: the important correlations ($T_d \rightarrow D_{2d}$) are $a_1 \rightarrow a_1$, $t_2 \rightarrow \{b_2+e\}$. The two electrons in the $3t_2$ orbital of $[Au_{12}Pb_{44}]^{8-}$ in Figure 7 are in fact located in the b_2 component of $3t_2$. The calculation is done in three stages. In the first, all eight valence orbitals of $[Au_8]^{4+}$ (right hand side) are included in the valence space of the fragment, so all of the interaction pathways are available. Then, the $2b_2$, 2e and $2a_1$ orbitals (those involved primarily in σ symmetry interactions) are removed from the valence space. The result is a reduction in the orbital interaction terms, primarily in the a_1, b_2 and e representations, and an overall loss of 2.03 eV in bonding energy. Further elimination of the 1e orbital (involved primarily in π-symmetry interactions) from the valence space results in a further loss of 2.70 eV in bonding energy, localized almost entirely in the e representation. The large residual bonding energy even after all the virtual orbitals of $[Au_8]^{4+}$ are removed arises from a redistribution of electron density between the occupied and virtual orbitals of the four $[AuPb_{11}]^{3-}$ fragments to maintain orthogonality. From this analysis, we conclude that the secondary π interactions between the $Au@Pb_{11}$ cluster units and the capping Au atoms are very significant from an energetic point of view.

Supplementary Table 8. Total interaction energies and their components (in eV) for the fragmentation of $[Au_{12}Pb_{44}]^{8-}$ into $([Au@Pb_{11}]^{3-})_4 + [Au_8]^{4+}$.

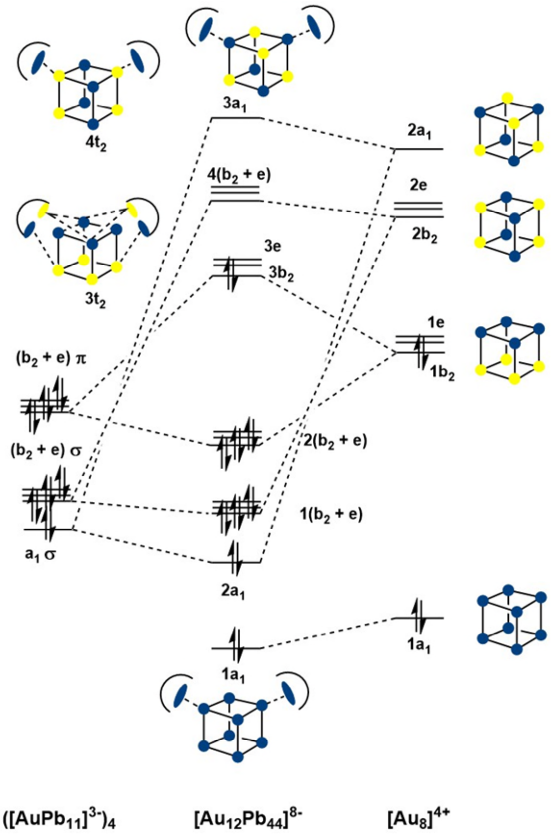

		All valence orbitals on $[Au_8]^{4+}$ included	{2b₂, 2e} and 2a₁ removed from $[Au_8]^{4+}$	1e, {2b₂, 2e} and 2a₁ removed from $[Au_8]^{4+}$
E_{steric}		–14.66	–14.66	–14.66
E_{orb}				
	a_1	–4.05	–3.68	–3.75
	a_2	–1.58	–1.64	–1.69
	b_1	–2.06	–2.14	–2.21
	b_2	–4.80	–4.05	–4.17
	e	–18.01	–16.92	–13.69
	total	–30.50	–28.43	–25.52
E_{solv}		–54.16	–54.20	–54.41
E_{tot}		–99.04	–97.01	–94.31

Nalewajski-Mrozek bond orders in $[Au@AuPb_{11}]^{3-}$ and $[Au_{12}Pb_{44}]^{8-}$

Supplementary Table 9. Nalewajski-Mrozek Bond orders

$[Au@AuPb_{11}]^{3-}$

The calculated bond orders (in the C_{5v}-symetrized $^1A_1$ state) can be broken down into distinct types:

Au-Au within the icosahedra.	0.07
Endohedral Au to Pb atoms of the icosahedron	0.08 (to the open face)
	0.18 (to the equatorial Pb_5 ring)
	0.24 to the Pb on the 5-fold axis
Surface Au to Pb atoms of the icosahedron	0.25

$[Au_{12}Pb_{44}]^{8-}$

The calculated bond orders (in the D_{2d}-symetrized $^3A_2$ state) can be broken down into distinct types:

Au-Au within the icosahedra.	0.05
Au-Au between atoms in the Au_8 core.	0.09-0.11
Endohedral Au to Pb atoms of the icosahedron	0.11 (to the open face)
	0.15 (to the equatorial Pb_5 ring)
	0.14 to the Pb on the 5-fold axis
Surface Au to Pb atoms of the icosahedron	0.17-0.21
Secondary Pb…Au interactions (< 3.7 Å)	0.07-0.12

Similar numbers are calculated for different spin states (^3E, $^3A_2$) and also for the triplet state of the X-ray geometry (unsymmetrized).

Supplementary References

[1] C. Croix, A. Balland-Longeau, H. Allouchi, M. Giorgic, A. Duchêne, J. Thibonnet, *J. Organomet. Chem.* **2005**, *690*, 4835-4843.

[2] E. M. Meyer, S. Gambarotta, C. Floriani, A. Chiesi-Villa, C. Guastinit, *Organometallics.* **1989**, *8*, 1067 – 1079.

[3] G. M. Sheldrick, *Acta Crystallogr. Sect. A: Found. Adv.* **2015**, *71*, 3-8.

[4] O. V. Dolomanov, L. J. Bourhis, R. J. Gildea, J. A. K. Howard, H. Puschmann, *J. Appl. Crystallogr.* **2009**, *42*, 339-341.

[5] A. L. Spek, *Acta Crystallogr., Sect. D: Biol. Crystallogr.* **2009**, *65*, 148-155.
